# The neuroprotective effect and RNA‐sequence analysis of postconditioning on the ischemic stroke with diabetes mellitus tree shrew model

**DOI:** 10.1002/brb3.2354

**Published:** 2021-09-24

**Authors:** Ling Zhao, Shufen Tan, Qiwei Liao, Xia Li, Tingyu Ke, Shuqing Li

**Affiliations:** ^1^ Department of Endocrinology The Second Affiliated Hospital of Kunming Medical University Kunming China; ^2^ Department of Gynecologic Oncology The Third Affiliated Hospital of Kunming Medical University Kunming China; ^3^ Department of Cardiology The Yan‐an Affiliated Hospital of Kunming Medical University Kunming China; ^4^ Department of Pathophysiology Kunming Medical University Kunming China

**Keywords:** diabetes mellitus, ischemic stroke, neuroprotective effect, RNA‐sequence analysis of postconditioning

## Abstract

**Introduction:**

Patients with comorbidity of ischemic stroke (IS) and diabetes mellitus (DM) show poor neurological functional recovery, and ischemic postconditioning (IPOC) should be considered a powerful neuroprotective method for IS. However, whether it should be introduced for patients with IS and DM remains controversial. This study established a DM with IS (DMIS) tree shrew model, which was intervened by IPOC to assess its neuroprotective effects and also to analyze the relevant mechanism by RNA‐sequence and bioinformatics analysis.

**Methods:**

Fifty‐four tree shrews were randomly divided into a sham operation control group, a DMIS group, and an IPOC group (DMIS model), with 18 tree shrews per group. Triphenyl tetrazolium chloride (TTC), hematoxylin‐eosin (HE) staining, transmission electron microscopy (TEM), and RNA‐sequence analysis were performed to assess the IPOC effect.

**Results:**

IPOC reduced infarct size and reduced nerve cell injury in IS tree shrews with DM. RNA‐seq analysis showed that IPOC significantly increased the expression of the homeobox protein SIX3, while downregulating the expression of HLA class II histocompatibility antigens DQ beta 1 chain, CAS1 domain‐containing protein 1, and cytokine receptor‐like factor 2. The most downregulated signaling pathways include the NF‐κB signaling pathway, TNF signaling pathway, and Fc gamma R‐mediated phagocytosis.

**Conclusions:**

IPOCs have a neuroprotective effect in a DMIS animal model that reduces infarct size and nerve cell injury. This mechanism might be related to reducing inflammation and stress responses that decreases the activity of TNF and NF‐κB signaling pathways.

## Graphical abstract

IPOC had a neuroprotective effect in a diabetic ischemic stroke model to reduce infarct size and nerve cell damage. Through RNA‐seq analysis, IPOC clearly downregulated the expression of HLA class II histocompatibility antigen DQ beta 1 chain, CAS1 domain‐containing protein 1, and cytokine receptor‐like factor 2. In addition, the downregulation of the NF‐?B signaling pathway, TNF signaling pathway, and Fc gamma R‐mediated phagocytosis indicate these may be the main pathways for IPOC intervention.

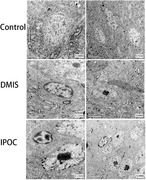

## 1 INTRODUCTION

The prevalence of ischemic stroke (IS) and diabetes mellitus (DM) is increasing with the aging population. IS is a serious threat to human health with a high fatality and disability rate, while DM is an independent risk factor for stroke (Shou et al., 2015). The risk of IS is 1.5 to 4 times higher in patients with DM than those without (Mitsios et al., 2018), furthermore, 36.6% of patients with strokes also had DM (Liu et al., 2016). Patients with both IS and DM are prone to abnormal lipid metabolism, hypertension, atherosclerosis, chronic inflammation, etc. (Hong et al., 2019). In addition, endothelial dysfunction caused by DM also increases thrombosis, monocyte activation, premature atherosclerosis, and vulnerable plaques (Olesen et al., 2019). Therefore, patients with IS and DM have larger infarct sizes, severe edemas, and relapse rates than non‐DM stroke patients, which affects the neurological recovery and prognosis (Akhtar et al., 2019; Huynh et al., 2017; Lau et al., 2019 ).

The protective effect of ischemic preconditioning has been determined against ischemia reperfusion injury. However, there are various difficulties that introducing ischemic preconditioning faces in clinical practice (Babiker, 2016). Ischemic postconditioning (IPOC) introduces intermittent sequential interruptions of the blood supply in the early stage of reperfusion after ischemia, which gives a similar effect to ischemic preconditioning (Vetrovoy et al., 2017). Hypoxia is a key destructive factor in the IS process. IPOC can increase the tolerance of cerebral hypoxia by reducing reactive oxygen species, the level of malondialdehyde, and the level of oxidized protein modification, while increasing the activities of superoxide dismutase and catalase (Fagova et al., 2019; Zhao et al., 2014 ). In addition, studies have shown that IPOC can protect nerve cell mitochondria by stabilizing voltage‐dependent ion channels (Yao et al., 2018), promoting the upregulation of anti‐apoptotic factors and neurotrophic factors such as HIF‐1 (Vetrovoy et al., 2017), and phosphorylation of the Akt signaling pathway (Liu et al., 2013) to provide a powerful neuroprotective effect for stroke.

However, the neuroprotective effect and mechanism of IPOC in patients with IS and DM still requires more comprehensive research. This study aims to establish a diabetic IS tree shrew model with IPOC intervention to explore the neuroprotective effect and analyze the relevant mechanism by RNA‐sequence and bioinformatics analysis.

## 2 MATERIALS AND METHODS

### 2.1 Animals and grouping

Healthy adult male tree shrews weighing 130 ± 30 g (SYXK (Dian) K2013‐0001, National Primate Research Center, Kunming, China) were used. The rearing conditions were artificial light for 12 hr, 25 ± 3°C, humidity 40% to 60%, and free eating and drinking. The entire experimental process was approved, supervised, and inspected by the Experimental Animal Ethics Committee of Kunming Medical University (kmmu2021099). Fifty‐four tree shrews were randomly divided into three groups with 18 in each group. They were the sham operation control group, the diabetes mellitus with ischemic stroke (DMIS) group, and the DMIS with IPOC intervention group.

### 2.2 DM model establishment

Before starting, animals were adaptively fed for 1 week. The tree shrews in the DMIS and IPOC groups were fed conventional feed to high‐fat feed (Chengdu Dashuo Experimental Animal Co., Ltd., Chengdu, China). The formula for high‐fat feed was basic feed, 1% cholesterol, 0.1% sodium cholate, 10% lard, 5% egg yolk powder, and 5% whole milk powder. The purpose of the high‐fat feed feeding was to induce insulin resistance with high fat and high cholesterol levels, and to make the model closer to the pathogenesis of clinical DM.

After 8 weeks of high‐fat feeding, 2% streptozotocin (STZ) dissolved in 0.01 mol/L citric acid‐sodium citrate buffer was injected into the femoral vein at a dose of 100 mg/kg body weight. The control group animals were injected with 0.01 mol/L citric acid‐sodium citrate buffer. After injection, the blood glucose level was higher than 16.7 mmol/L at three time points (3, 7, and 14 days). This was considered a successful level for establishment of the DM model that then maintained hyperglycemia for 4 weeks.

### 2.3 Thrombotic cerebral IS model establishment

The tree shrews of the DMIS and IPOC groups were anesthetized, fixed, and subjected to skin disinfection. A 1 cm incision was made at the midpoint of the connection between the right tragus and the lateral canthus of the right eye. The temporal muscle was separated to expose the skull. A sterile aluminum sheet with 0.5 cm diameter hole in the center was inserted into the incision. The red bengal physiological saline solution (1.33 mL/kg) was injected into the femoral vein, and the SQ‐III cerebral thrombosis device (patent number: ZL201420068737.2) was used 10 min after injection. A special light beam with a 560 nm central wavelength and 1.0 W/cm^2^ light intensity was passed through the hole of the sheet and into the skull. This would react with the red bengal solution and oxygen in the cerebral blood vessels to damage the vascular endothelium and induce thrombosis (Zhao et al., 2019). The irradiation time was 15 min. The tree shrews in the control sham group were also subjected to the surgical operation, however normal saline was injected and no light was given.

### 2.4 IPOC intervention

After the establishment of the IS, the IPOC was implemented in the 4‐hr time period by intermittently and repeatedly clamping the common carotid artery on the ischemic side. The common carotid artery was clamped for 5 min with a non‐invasive arterial clip on the upper edge of the thyroid cartilage, and then the clip was removed for 5 min for three cycles. Tree shrews in the DMIS and control groups were separated from the right common carotid artery without clipping.

### 2.5 Laboratory blood test

Nine animals were randomly selected from each group 24 hr after IS. After anesthesia, blood was collected from the abdominal aorta into a coagulation tube. The blood was centrifuged at 3000 r/min for 10 min, and serum was collected and stored in a cryotube at −80°C. The test indicators included total protein, albumin, globulin, albumin/globulin, alanine aminotransferase, aspartate aminotransferase, urea, creatinine, uric acid, blood lipids, blood glucose, etc.

### 2.6 Triphenyl tetrazolium chloride staining

Five tree shrews were randomly selected from each group. After blood collection, the animals were sacrificed. The skull was quickly opened with rongeurs, and the whole brain was removed. Whole brain tissue was placed in 2% triphenyl tetrazolium chloride (TTC) solution and incubated in a 37°C water bath for 30 min in the dark. Staining pictures were taken with a digital camera and the percentage of the cortical infarct area in the half‐brain section was calculated using MiVnt Microsoft 2.0 image analysis software.

### 2.7 Hematoxylin‐eosin staining

Five models were randomly selected from each group. After anesthesia, the heart was exposed, and the descending aorta was ligated. Then, the right atrial appendage was cut, and 100 mL of 4°C normal saline and 200 mL of 4°C 10% neutral buffered formaldehyde was injected into the left ventricle. The whole brain was removed and placed in 10% neutral buffered formaldehyde. The ischemic part of the brain (same corresponding part in the control group) was embedded into paraffin and cut into μm sections and stored at 4°C. These were then used for hematoxylin‐eosin (HE) staining and observed under a light microscope.

### 2.8 Transmission electron microscopy

Five models were randomly selected from each group and were quickly sacrificed after anesthesia to obtain the ischemic cortex tissue. Infarct tissue was cut into 1 × 1 × 1 mm pieces and fixed in 3.5% glutaraldehyde at 4°C. After washing, the specimen was placed in 1% tetraoxosinic acid for 24 h to ensure gradient dehydration, propylene oxide infiltration, embedding, and ultrathin sections. Double staining was performed with lead citrate and uranyl acetate and the ultrastructure of the cerebral cortex was observed with a JEM‐1011 transmission electron microscope.

### 2.9 RNA‐sequence analysis

Three tree shrews were randomly selected from each group and quickly sacrificed after anesthesia. Brain tissues were obtained on ice. After washing with 0.9% normal saline, the ischemic nerve tissue was clamped and stored at −80°C. Total RNA was extracted according to the kit manufacturer's instructions. In detail, the tissue was homogenized after adding TRIzol, centrifuged at 12,000 rpm for 10 min at 4°C, and the supernatant was collected. Add chloroform to lyse, centrifuge at 12,000 rpm at 4°C for 15 min, and then the supernatant was collected. After adding 70% ethanol, it was mixed well and purified by adsorption. A total RNA sample was obtained by centrifugation and rinsing. The total amount of each sample was 3 μg of RNA, and the sequencing library was constructed according to the NEMBext Ultra TM RNA LibraryPrep kit. To extract mRNA, this was used as a template to synthesize first‐strand cDNA and second‐strand cDNA, and these were purified to obtain a cDNA library. RNA sequencing was performed using Illumina Hi‐seq2500.

### 2.10 Differential gene expression and bioinformatics analysis

All samples were checked for cross‐species contamination and compared with the tree shrew genome. The spliced unigenes were also compared with public gene data and functional homology annotation was performed. Gene differential expression analysis was performed among samples from each group, including fold change analysis, Fisher's tests, and Chi‐square tests. The top 20 differentially expressed genes were analyzed by gene ontology (GO) enrichment analysis and the Kyoto Encyclopedia of Genes and Genomes (KEGG) pathway analysis. GO analysis included three categories: biological process, cellular component, and molecular function.

### 2.11 Statistical analysis

The experimental data conforming to the normal distribution were expressed as mean ± standard deviation (m ± s); otherwise, the median and interquartile range were used. One‐way analysis of variance was used for multi‐group comparison, and the pairwise comparison between groups was performed using the q test. *P*‐values less than 0.05 indicated the difference was significant. SPSS 17.0 statistical software was used in analysis.

## 3 RESULTS

### 3.1 General observation and biochemical indicators

The tree shrews body weights were not significantly different in each group before the model was established. After model induction, the water intake and urine volume of tree shrews in the DMIS and IPOC groups were significantly increased compared to the control group. The body weights of the DMIS and IPOC groups were also significantly reduced compared to the control. For blood glucose levels, tree shrews in the DMIS and IPOC groups were significantly higher than in the control group. In addition, the blood glucose levels of the IPOC group were significantly lower than the DMIS group (two sample *t*‐test, *P *= 0.0017). This indicated that IPOC intervention may have a protective effect on diabetic tree shrews and help to alleviate the large fluctuations in blood glucose levels. For the other biochemical indicators; the lactate dehydrogenase isoenzyme 1(LDH1, *P *= 0.0345), cholesterol (CHOL, *P *= 0.0078), low‐density lipoprotein cholesterol (LDL‐CH, *P *= 0.0423), triglyceride (TG, *P *= 0.0095), and C‐reactive protein (CRP, *P *= 0.0498) were all significantly higher in the DMIS and IPOC groups than in the control group. Total bilirubin (TB) in the control group was significantly higher than in the other groups (*P *= 0.0014). There was no significant difference in the other indicators among the groups (Table 1).

**TABLE 1:** Comparison of body weight and blood biochemical indexes in tree shrews (Mean± SD)

Indexes	Control group	DMIS group	IPOC group	F	*P value*
Initial Body weight	138.97±9.61	134.32±15.73	135.43±14.74	0.304	0.587
Body weight after stroke	141.15±11.76	120.29±13.82	117.12±12.68	15.91	<0.001
Total protein	52.96±3.62	53.93±3.26	53.96±2.87	0.422	0.522
ALB	35.93±2.99	35.66±4.22	42.51±12.46	3.212	0.0857
ALT	207.88±289.94	117.78±44.58	154.00±124.21	0.386	0.54
GLOB	21.93±7.59	23.21±7.34	26.53±11.49	1.173	0.29
Cr	14.00±6.75	17.75±8.57	17.00±4.95	0.847	0.367
UREA	13.00±7.74	11.96±3.99	13.11±7.10	0.001	0.972
GLU	5.38±0.52	5.35±1.09	5.78±1.09	0.816	0.375
GLU after DM modeling	6.52±1.32	32.29±6.08	23.04±4.13	66.07	2.38E‐08
UA	23.32±12.77	13.71±11.60	14.20±6.48	3.306	0.0815
LDH1	6.97±3.99	24.49±17.22	20.46±13.28	5.026	0.0345
CK‐MB	42.50±15.78	136.00±161.30	79.63±52.48	0.641	0.431
LDH	318.50±107.67	437.50±206.40	376.63±113.58	0.68	0.418
HBDH	356.50±90.40	573.88±254.82	513.25±220.79	2.722	0.112
CK	223.13±51.73	264.38±79.54	239.38±86.36	0.217	0.646
TB	19.60±8.15	12.59±4.31	10.21±1.98	13.39	0.00124
AST	252.09±130.15	277.61±105.02	191.12±80.70	1.455	0.239
CHOL	2.85±0.73	7.80±3.41	6.53±3.08	8.445	0.00775
HDL‐CH	1.84±0.40	1.50±0.53	1.93±0.40	0.182	0.673
LDL‐CH	1.53±0.37	3.06±0.95	2.32±0.89	4.6	0.0423
TG	1.02±0.45	1.73±0.29	1.66±0.64	7.943	0.00952
CRP	0.51±0.24	1.43±0.53	0.98±0.60	4.269	0.0498
TPT	1.67±1.87	0.77±0.52	0.73±0.66	2.838	0.105
MYO	63.91±53.90	45.86±21.80	41.21±13.80	1.948	0.176

### 3.2 TTC staining

Twenty‐four hours after photoreaction induced thrombotic cerebral ischemia, there were no abnormal changes in the brain tissue of the control group in general observation. In the DMIS and IPOC groups, obvious ischemic edema areas were observed. After TTC staining, no obvious infarcted areas were seen in the control group. The DMIS group had an obvious infarct area, accounting for 19.56 ± 1.25% of the half‐brain. In the IPOC group, the infarct size was smaller than that in the DMIS group at 15.12 ± 2.13%. The results indicated that IPOC might have a neuroprotective effect on diabetic cerebral infarction, which has already been addressed in our previous study (Zhao et al., 2018; Figure S1).

### 3.3 HE staining

The brain tissue of the control group had a complete and distinct cortex structure, normal cell morphology, rich cytoplasm, and obvious nucleoli. Furthermore, no nuclear pyknosis was observed. However, in the DMIS group, the nerve cells in the ischemic area were pyknotic with gaps around the cells. The cell arrangement was loose, and many remaining cells lost their complete cell structure. In the IPOC group, the ischemic areas cortical tissue still had normal nerve cell morphology, and these cells were arranged neatly. The overall degree of damage was less than that of the DMIS group (Zhao et al., 2018; Figure S1).

### 3.4 TEM results

**FIGURE 1:**
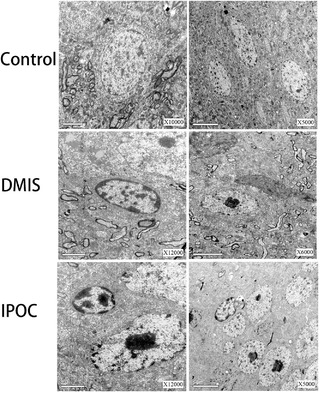
Transmission electron microscopy (TEM) was used to observe the changes of neural ultrastructure in ischemic cortical areas

In the control group, the cortex nerve cells had a normal morphology and complete structure. The cell membrane was clear, the mitochondria had normal morphology, there was regular endoplasmic reticulum arrangement, and rough endoplasmic reticulum ribosomes were clear and complete. The nuclei, membranes, and nucleoli were also clear. The cortexes of the DMIS group showed obvious ischemic damage, and a large number of nerve cells had undergone pyknosis, with irregular nuclei and increased heterochromatin. The structure of the organelles in the cytoplasm was unclear. The mitochondria cristae were swollen and they were dissolved, forming parts of vacuoles. The endoplasmic reticulum swelled and expanded to form the endoplasmic reticulum pool. In the IPOC group, a small number of nerve cells underwent pyknosis. The mitochondria found in the cortex of the ischemic area were swollen and some cristae disappeared. The endoplasmic reticulum arrangement was normal. The nucleus was irregular, its membrane was uneven, and the nucleolus was enlarged (Figure 1).

### 3.5 Quality control of the RNA‐sequence

In this study, polyadenylated RNA was captured to analyze the transcriptome of ischemic cerebral tissue. Nine sequencing libraries were prepared. The clean bases produced in each group were as follows: control group (sham 1, sham 2, sham 3): 21.09 GB; DMIS group (DM + IS 1, DM + IS 2, DM + IS 3): 21.81 GB; IPOC group (DM+IS+PC 1, DM+IS+PC 2, DM+IS+PC 3): 20.4 GB. After removing the low‐quality readings, a clean reading of more than 95% was harvested at Phred‐scale quality Q20, and more than 90% at Q30 level. The GC content was about 50%.

### 3.6 Gene expression level

**FIGURE 2:**
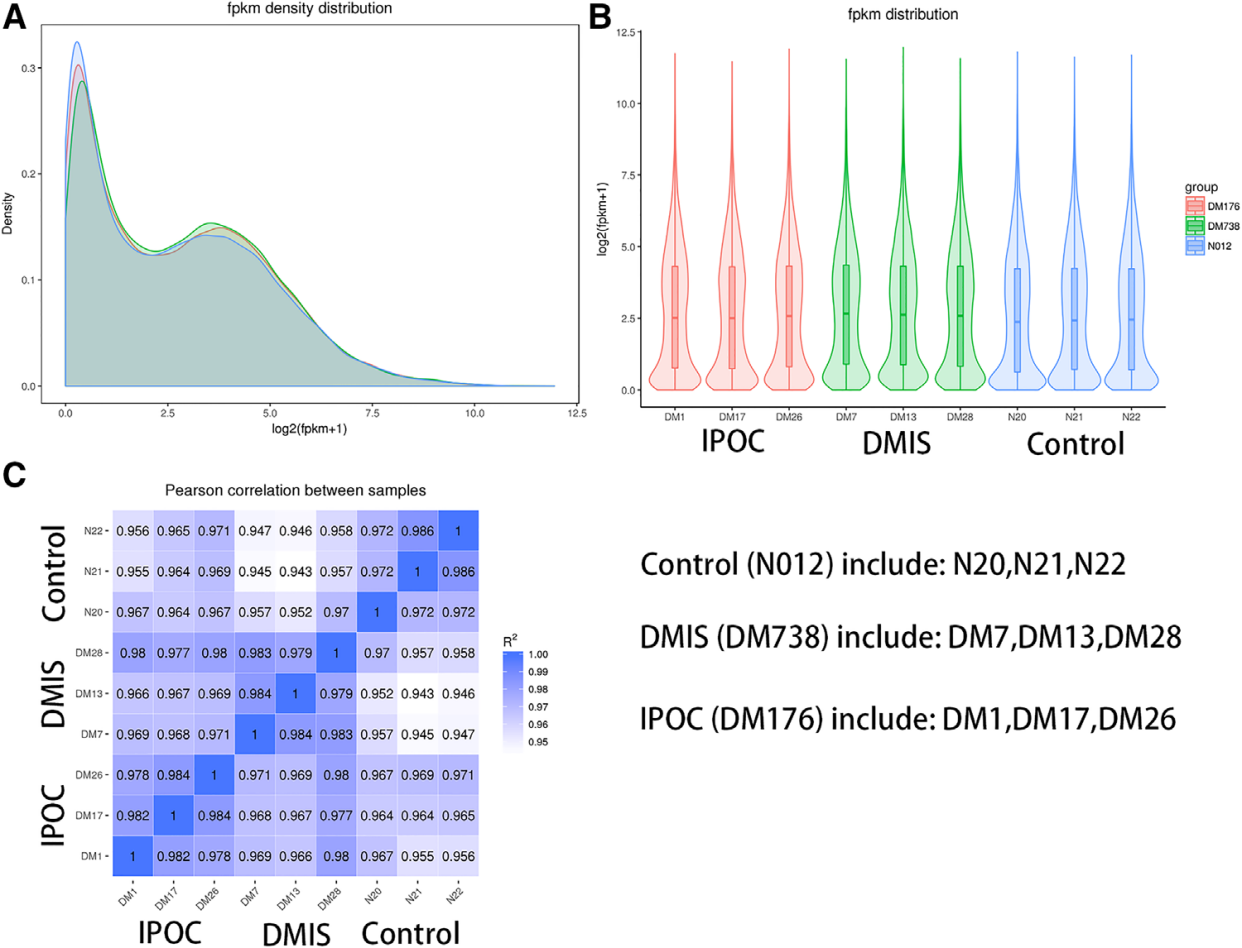
Comparison of gene expression levels in each group with FPKM distribution and biological repeat correlation tests. A: FPKM density distribution plot; B: violin plot of FPKM distribution; C: RNA‐Seq correlation tests

In the study, the FPKM distribution plot (Figure 2a) and violin plot (Figure 2b) were used to compare gene expression levels among groups. The results showed that there were no differences in gene density distribution amongst the groups, and that the overall distribution was consistent. In addition, this study carried out three biological replicates. The RNA‐Seq correlation test results showed that the grouping was reasonable and that the biological reproducibility in each group was good (Figure 2c).

### 3.7 Differentially expressed genes

The gene expression profile between the DMIS group and the IPOC group revealed 520 differentially expressed genes with 203 upregulated genes and 317 downregulated genes. The top three upregulated expressed genes in the DMIS group included Novel01439, homeobox protein SIX3, and uncharacterized protein LOC105490759 isoform X1. The most downregulated expressed genes included HLA class II histocompatibility antigen DQ beta 1 chain, CAS1 domain‐containing protein 1, and cytokine receptor‐like factor 2 (Table 2).

**TABLE 2:** The top 3 up‐regulated and down‐regulated differential expression genes between DMIS and IPOC group

Gene_id	log2FoldChange	*p*adj	Blast swiss prot
Novel01439	5.495	0.002432	‐//‐
102483157	4.8801	0.0036411	Homeobox protein SIX3
Novel00930	3.9866	5.02E‐35	uncharacterized protein LOC105490759 isoform X1
102503204	−6.5808	0.010365	HLA class II histocompatibility antigen, DQ beta 1 chain
102476790	−4.859	0.013696	CAS1 domain‐containing protein 1
102476854	−4.2486	0.0069143	Cytokine receptor‐like factor 2

### 3.8 GO enrichment

**FIGURE 3:**
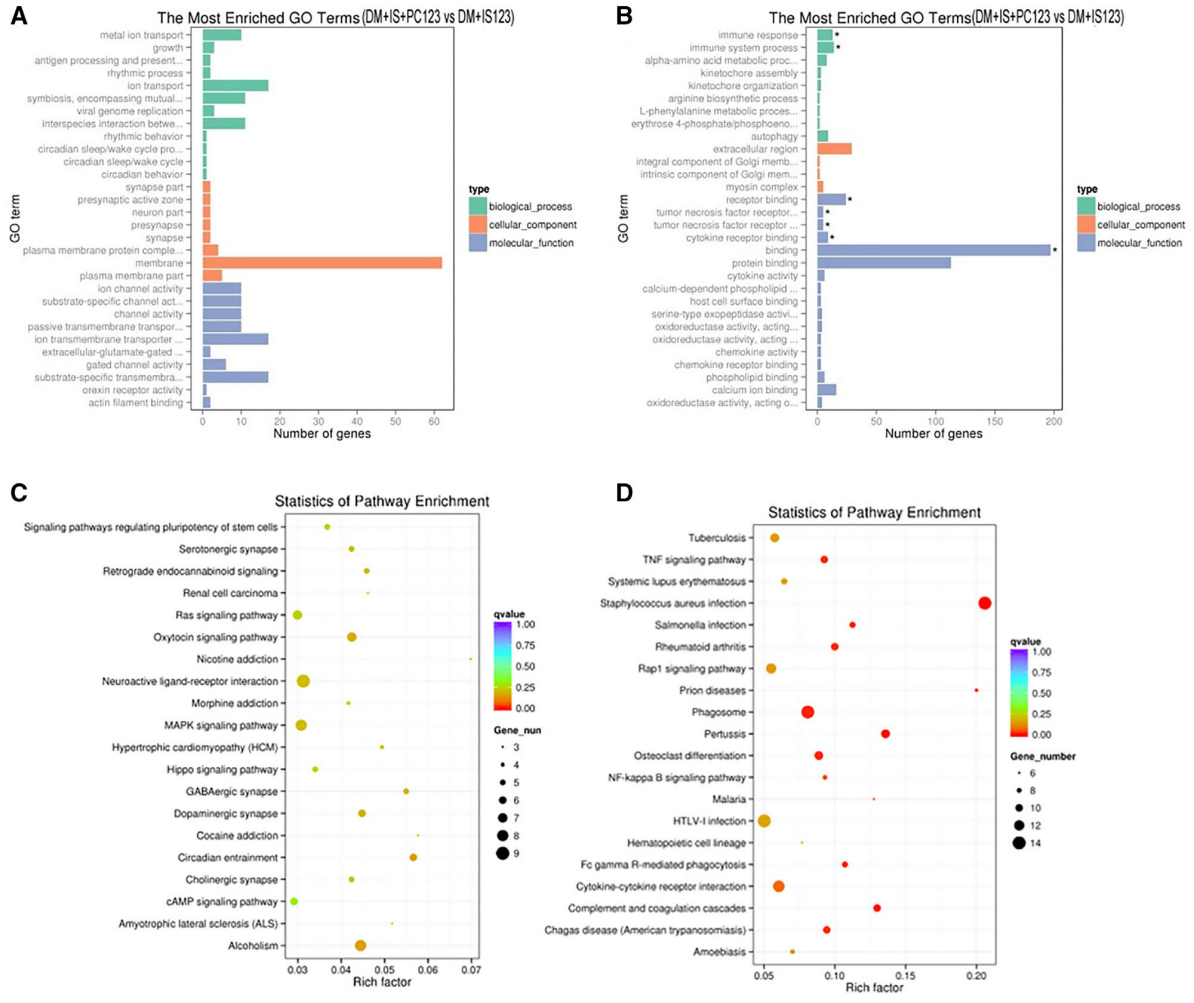
Gene ontology (GO) enrichment analysis and Kyoto Encyclopedia of Genes and Genomes (KEGG) signaling pathway analysis of differentially expressed genes in each group. The GO analysis of upregulated genes (a) and downregulated genes (b) between the DMIS and IPOC groups; the KEGG signaling pathway analysis of upregulated genes (c) and downregulated genes (d) between the DMIS and IPOC groups

For GO enrichment analysis, there was no significant differences in the upregulated genes of biological process (BP), cellular component (CC), and molecular function (MF) classifications between the IPOC and DMIS groups (*p*_adj_ > 0.05; Figure 3a). The significantly downregulated genes in the BP classification were mainly in the immune system process (*p*_adj_ < 0.05), these were receptor‐type tyrosine‐protein phosphatase C (PTPRC), TNF13, and CXCL2. The immune responses (*p*_adj_ < 0.05) main genes involved were C1QB, CCL5, and CCAAT/enhancer‐binding protein delta (CEBPD). There was no significant differences in downregulated genes that existed in the CC classification (*p*_adj_ > 0.05). The downregulated genes in the MF classification were mainly in receptor binding (*p*_adj_ > 0.05), which involved TNF14, C1QA, and IL‐6; tumor necrosis factor receptor binding (*p*_adj_ < 0.05), which involved C1QA, TNF14, and TNF13; and cytokine receptor binding (*p*_adj_ < 0.05), which involved TNF14, CXCL2, and C1QA (Figure 3b).

In the KEGG analysis, the IPOC group and the DMIS group had 0 differential signaling pathways for upregulated genes (*p*_adj_ > 0.05; Figure 3c). However, there were 13 differential signaling pathways for downregulated genes (*p*_adj_ < 0.05), mainly involving the NF‐κB signaling pathway, TNF signaling pathway, and Fc gamma R‐mediated phagocytosis (Figure 3d).

## 4 DISCUSSION

Patients with DM had a relatively higher risk of stroke, larger infarct size, higher recurrence and mortality, and poorer neurological recovery after stroke compared to those without. This suggests that IS when combined with DM has a different pathological development process, for example, hyperglycemia affects the integrity of white brain matter and alters the production of oligodendrocytes, which reduces neuroplasticity. However, there was still no difference in the clinical treatment strategy of patients with strokes with or without DM, except for hypoglycemic therapy. Therefore, it was necessary to classify patients with IS and DM separately from those without DM and conduct research on related intervention mechanisms.

IPOC is a feasible neuroprotection method after IS, but there is still a lack of research on whether it is effective in the DMIS model. A prior study found the expression of γ‐enolase, thioredoxin, and protein phosphatase 2A subunit B was lower, and the expression of collapsin response mediator protein 2 and HSP60 in the striatum was elevated after ischemic injury (Shah et al., 2018). Moreover, there were significant differences among transient IS, permanent IS, and permanent DMIS models for key signaling molecules involved in neuronal apoptosis, glutamate induced excitotoxicity, neuroinflammation, oxidative stress, and neurotrophic changes (Shah et al., 2019). In this study on the tree shrew DMIS model, IPOC was found to reduce infarct size and reduce nerve cell injury. Further RNA‐seq analysis showed that IPOC significantly increased the expression of the homeobox protein SIX3, while it downregulated the expression of the HLA class II histocompatibility antigen, DQ beta 1 chain, CAS1 domain‐containing protein 1, and cytokine receptor‐like factor 2. The GO annotation showed that the BPs were mainly involved in the immune system and response, whereas MFs were involved in receptor binding, tumor necrosis factor receptor binding, tumor necrosis factor superfamily receptor binding, and cytokine receptor binding. The most downregulated signaling pathways include the NF‐κB signaling pathway, TNF signaling pathway, and Fc gamma R‐mediated phagocytosis.

IPOC has shown a protective effect on infarct size and neurological deficits in basic and clinical studies of IS. A meta‐analysis showed that IPOC may provide brain protection after stroke that can significantly reduce the recurrence of stroke and transient ischemic attacks (TIA), while also reducing the NIHSS score, modified Rankin Scale, and highly sensitive CRP. The main mechanisms involved improving cerebral perfusion, which protects the integrity of the blood‐brain barrier, and enhances the collateral circulation (Che et al., 2019; Li et al., 2018; Li et al., 2018; Zhang et al., 2019 ). In addition, the mTOR signaling pathway may also play an important role in the protective mechanism (Wang et al., 2018). It should be noted in the clinical application that the regimen of specific IPOCs may affect the final protective effect, such as the number of cycles, duration in each cycle, and total intervention time (Lee et al., 2018).

For the protective effect of IPOC on the DMIS model, basic research has confirmed that limb remote IPOC can reduce brain damage and neurological deficits, decrease the infiltration of CD8^+^ T cells, and increase the number of B cells recruited to the ischemic site. Remote IPOC also increased the expression of p‐ERK in the ipsilateral hemisphere of the ischemia and attenuated the level of IL‐6 in peripheral blood (Liu et al., 2020). This study used the common carotid artery ligation IPOC method, which may have a more direct intervention on the ischemic brain than remote IPOC. However, the difference in the effect between these two methods still needs to be confirmed by further experiments.

Meanwhile, this study found that IPOC reduces the activity of TNF and NF‐κB signaling pathways, which are also related to reducing inflammation and stress responses. In other research, remote IPOCs can also reduce the upregulation and activation of TNF‐α and NF‐κB, caused by cerebral ischemia‐reperfusion injury, and reduce the infarct size and the number of neuronal apoptosis (Cheng et al., 2014). The neuroprotective agent Nobiletin also has anti‐inflammatory properties by reducing the expression of TNF‐α and downregulating the NF‐κB pathway (Zheng et al., 2017).

In addition, this study also found that cytokine receptor‐like factor 2 expression is obviously downregulated after IPOC. This gene could form a functional complex with IL‐7R and stimulate cell proliferation via the active STAT3/STAT5 pathway. Relevant studies have shown that the STAT3 pathway plays a central role in the activation of microglia and the neuroinflammatory response induced by elevated plasma homocysteine. Thus, the STAT3 inhibitor, AG490, could inhibit the phosphorylation of STAT3, microglia activation, and secretion of IL‐6 and TNF‐α to produce neuroprotective effects (Chen et al., 2017; Cheng et al., 2019; Li et al., 2019 ). This study also suggests that cytokine receptor‐like factor 2 may serve as a new target for inhibiting the STAT3 signaling pathway in DMIS treatment. However, there are still opposing opinions on the role of the STAT3 signaling pathway in nerve recovery, suggesting that STAT3 upregulation is related to the promotion of angiogenesis, neurogenesis, and function recovery after IS (Chen et al., 2018; Hou et al., 2018 ). These different results may be related to the cell types detected, such as neurons and microglia, and the time points after stroke, such as the acute and chronic phases.

Finally, regarding the choice of animal models, in recent years, due to the exhaustion of non‐human primate resources and ethical issues, there is a new challenge in selecting economical and alternative primate laboratory animals. This study used tree shrews as experimental animals, as they are much more similar to humans in terms of biological characteristics, metabolism, physiology, and genomes when compared to rodents. The skulls of the tree shrews are thin at only one‐third the thickness of rats, which is conducive to the photochemical reaction of the special light beam to cause vascular endothelial damage, platelet adhesion/aggregation, and thrombus formation. Since the DMIS model does not require a craniotomy with a low infection rate, which is convenient for long‐term observation (Yao et al., 2017; Gu et al., 2020).

## 5 CONCLUSIONS

IPOC had a neuroprotective effect in a diabetic ischemic stroke model to reduce infarct size and nerve cell damage. Through RNA‐seq analysis, IPOC clearly downregulated the expression of HLA class II histocompatibility antigen DQ beta 1 chain, CAS1 domain‐containing protein 1, and cytokine receptor‐like factor 2. In addition, the downregulation of the NF‐κB signaling pathway, TNF signaling pathway, and Fc gamma R‐mediated phagocytosis indicate these may be the main pathways for IPOC intervention.

## CONFLICTS OF INTEREST

The authors have declared no conflict of interest.

## AUTHOR CONTRIBUTIONS

Ling Zhao and Tingyu Ke were associated with study concept and design, analysis and interpretation of data, drafting of the manuscript, and funding acquisition. Ling Zhao, Shiying Huang, and Qiwei Liao were associated with the acquisition of samples or data, statistical analysis, review of the manuscript. Ling Zhao, Xia Li, Shufen Tan, and Shuqing Li were associated with statistical analysis, review of the manuscript, material supports.

### PEER REVIEW

The peer review history for this article is available at https://publons.com/publon/10.1002/brb3.2354

## Supporting information

Supporting information.
Supplementary Figure 1. Hematoxylin‐eosin and triphenyl tetrazolium chloride staining results showed the cerebral cortex infarct size at 24 h after cerebral ischemia in each group of tree shrews.

## Data Availability

The datasets generated during and/or analysed during the current study are available from the corresponding author on reasonable request.
