# Supplementary figures and images for: The neuroprotective effect and RNA‐sequence analysis of postconditioning on the ischemic stroke with diabetes mellitus tree shrew model
Source: Brain Behav. 2021 Sep 24;11(11):e2354. doi: 10.1002/brb3.2354 (PMC8613421; doi:10.1002/brb3.2354)

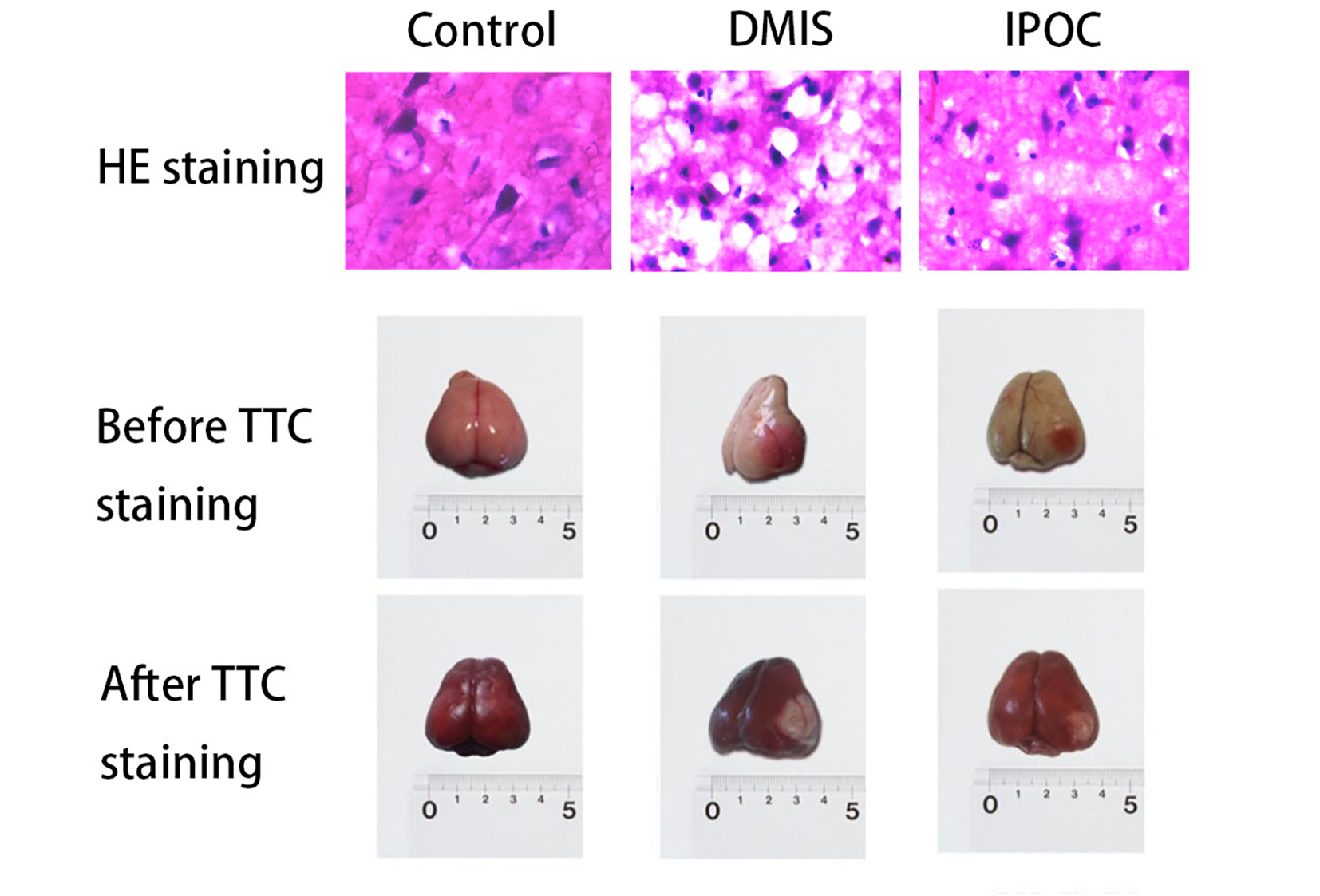

Supplement: Supplementary file 1 — Supporting information. Supplementary Figure 1. Hematoxylin‐eosin and triphenyl tetrazolium chloride staining results showed the cerebral cortex infarct size at 24 h after cerebral ischemia in each group of tree shrews. [file BRB3-11-e2354-s001.tif]
